# Complete Genome Sequence of GII.4 Human Norovirus HS191

**DOI:** 10.1128/genomeA.00169-12

**Published:** 2013-02-14

**Authors:** Stanislav V. Sosnovtsev, Karin Bok, Qiuhong Wang, Linda J. Saif, Kim Y. Green

**Affiliations:** a Laboratory of Infectious Diseases, National Institute of Allergy and Infectious Diseases, National Institutes of Health, Bethesda, Maryland, USA; b Food Animal Health Research Program, Ohio Agricultural Research and Development Center, Department of Veterinary Preventive Medicine, The Ohio State University, Wooster, Ohio, USA

## Abstract

Noroviruses are a common cause of gastrointestinal disease in humans worldwide. Here, we report the full-length genomic characterization of GII.4 norovirus strain HS191, which was associated with gastroenteritis in a laboratory worker in 2004.

## GENOME ANNOUNCEMENT

Noroviruses are a major cause of acute nonbacterial gastroenteritis outbreaks in humans. Their antigenic diversity is linked to variation in the capsid protein (VP1) gene. Based on the sequence of the VP1 gene, noroviruses are classified into five genogroups (GI to GV) and more than 30 genotypes. The GI, GII, and GIV strains have been shown to infect humans and, in some cases, animals, while the GIII and GV strains have been detected only in animals (1). Over the past two decades, the rapidly evolving noroviruses of the GII.4 genotype have emerged as major epidemic strains causing, in some years, up to 80% of all norovirus outbreaks (2, 3).

The GII.4 norovirus strain HS66 was associated with diarrhea in a child at Children’s Hospital in Columbus, OH, in 2001 and has been characterized extensively in the gnotobiotic piglet model (4). In 2004, while preparing new stocks of the HS66 virus, a laboratory worker was infected and developed gastroenteritis. Here, we report the full-length genome sequence of this virus, designated HS191. Viral RNA was extracted from diarrheic stool samples using an RNeasy mini kit (Qiagen). The extracted RNA was used as a template for the reverse transcriptase PCR (RT-PCR) amplification of cDNA fragments that overlap across the entire virus genome. The amplified cDNA fragments were sequenced using a set of norovirus genome-specific primers and an automated sequencer, ABI 3730 (Applied Biosystems). The sequences of the 5′- and 3′-end regions of the virus genome were determined using Rapid Amplification of cDNA Ends (RACE) system kits (Invitrogen). The full-length virus genome sequence was assembled using the Sequencher 4.9 program (GeneCodes).

The complete genome sequence of HS191 strain was 7,556 nucleotides (nt) in length, excluding the poly(A) tail. It contained three open reading frames (ORFs), ORF1, ORF2, and ORF3, with lengths of 5,100, 1,620, and 807 nt, respectively. The short 5′- and 3′-end nontranslated sequences were 4 nt and 46 nt long, respectively.

The nucleotide sequence of the HS191 VP1 gene (ORF2) was found to be identical to that of HS66 (GenBank accession no. EU105469), confirming the laboratory-acquired infection. A BLAST search of GenBank showed that the HS191/HS66 strain was most related to Hiroshima/19/2001 (GenBank accession no. AB504306) and VA98387/1998 (GenBank accession no. AY038600) strains, with levels of ORF2 nucleotide and deduced VP1 amino acid sequence identities reaching 98% and 99%, respectively.

A BLAST search of GenBank with the complete HS191 genome sequence revealed the highest identity (97%) with two GII.4 variants, strains Yuri 32073 and Dresden174/1997 (GenBank accession no. AB083781 and AY741811, respectively). Consistent with this, the HS191 virus clustered together with Yuri 32073 and Dresden174/1997 strains in the neighbor-joining phylogenetic tree inferred from a multiple norovirus genome sequence alignment.

A comparison of the full-length genome sequence of HS191 with that of HS66 will be important to determine whether adaptive mutations may have occurred in other regions of the genome during this transmission event. These data will be useful also for the comparative analysis of HS66 evolution in the gnotobiotic piglet model. The identification of adaptive mutations in nonhuman hosts may facilitate the further development and optimization of animal models for the study of human norovirus disease.

### Nucleotide sequence accession number.

The genome sequence of the HS191 isolate has been deposited in GenBank under the accession no. KC013592.
